# The association between the degree of cervical dilatation before ultrasound and physical examination indicated cerclage and subsequent neonatal outcomes

**DOI:** 10.5339/qmj.2024.20

**Published:** 2024-04-04

**Authors:** Ümran Kılınçdemir Turgut, Ebru Erdemoğlu, Cem Dağdelen, Osman Gürdal, Mehmet Okan Özkaya, Mekin Sezik

**Affiliations:** 1 University of Health Sciences, Adana City Training and Research Center, Department of Obstetrics and Gynaecology-Perinatology, Adana, Turkey Email: umrankilincdemiritf@hotmail.com; 2 Department of Obstetrics and Gynecology, Suleyman Demirel University Faculty of Medicine, Isparta, Turkey; 3 Department of Biostatistics and Medical Informatics, Medical School, Süleyman Demirel University, Isparta, Turkey

**Keywords:** cervical dilatation, emergency cerclage, McDonald cerclage, neonatal outcomes, preterm delivery

## Abstract

Introduction: Preterm identification of cervical dilation in pregnant women leads to the application of emergency cervical cerclage with an expectation of achieving term delivery. However, this is not always feasible. Short- and long-term neonatal complications post-preterm birth pose a significant challenge. It is crucial to anticipate potential complications and understand the possibilities of postpartum development as they can be encountered.

We aimed to evaluate the effect of the degree of cervical dilatation before ultrasound and physical examination-indicated cerclage in singleton pregnancies presenting with premature cervical dilatation with bulging fetal membranes (rescue cerclage) on subsequent neonatal outcomes.

Materials and Methods: In this retrospective clinical study, over a 10-year period between January 2009 and January 2019, 72 singleton pregnancies undergoing rescue cerclage were included and divided into two groups according to pre-cerclage cervical dilatation: Group 1 (n = 33) and Group 2 (n = 39) with cervical dilatation ≤3 cm and >3 cm, respectively. Latency period for pregnancy prolongation, gestational age at delivery, birth weight, and neonatal morbidity and mortality were compared across the groups. Logistic regression was used to delineate the independent effect of cervical dilatation at cerclage placement on neonatal mortality.

Results: Group 2 had a higher delivery rate at ≤28 weeks’ gestation (p = 0.007) and lower birth weight (p = 0.002) compared to Group 1, with an increased mean latency period in Group 2 (90 ± 55 days versus 52 ± 54 days, p = 0.005). The newborn intensive care unit (NICU) requirement, respiratory distress syndrome (RDS), neonatal jaundice and sepsis, and retinopathy of prematurity (ROP) were more frequent in Group 2. Neonatal mortality rate was higher (52.6% versus 24.2%, p = 0.015) and intact survival was lower (23.1% versus 48.4%, p = 0.013) in Group 2, whereas rates of cerebral palsy (8% and 9%, respectively) were similar between the groups (p = 0.64).

Conclusion: Advanced cervical dilatation (>3 cm) during physical examination-indicated cerclage in singleton pregnancies is associated with earlier delivery, leading to increased neonatal morbidity and mortality when compared with pregnancies having lesser degrees of cervical dilatation at cerclage. However, short-term poor neurological outcomes seem comparable.

## Introduction

Preterm labor is the start of labor before the 37th gestational week. Despite the use of antibiotics, cerclage application, prenatal steroid administration, and progesterone treatment, preterm labor continues to be a serious obstetric problem.^1^ Long-term morbidity due to preterm birth is a concern for surviving babies, and the frequency of morbidity increases as the gestational week decreases.^2^

Different surgical techniques for cervical cerclage application are described in the literature. The McDonald technique is one of the most commonly used techniques today.^3^ This technique involves a simple purse-string stitch applied around the cervix via a transvaginal approach.

Prophylactic cervical cerclage is applied to those who have a history of premature birth or pregnancy loss due to cervical insufficiency. The main indication for emergency (salvage) cervical cerclage is the presence of cervical effacement or detection of cervical dilatation before the 28th gestational week. Contraindications for emergency cervical cerclage include uterine contractions, rupture of fetal membranes, major fetal anomaly, unexplained vaginal bleeding, intrauterine/vaginal infection, and gestational age of >28 weeks.^4^

Poor neonatal outcomes have been reported after emergency cerclage application. Cerclage application in cases where the amniotic membrane prolapsed into the vagina caused an increase in the risk of cerebral palsy.^5^ A correlation between the increase in cervical dilatation before the cerclage procedure and poor pregnancy outcomes has been reported.^6^ In another study, cervical dilatation of ≥ 2 cm determined in manual examination before the cerclage procedure was associated with earlier delivery time independent of all other variables.^7^

Although many studies focus on neonatal outcomes in pregnant women who underwent emergency cervical cerclage, data on the postnatal infancy and childhood periods are limited.

This study aims to evaluate the effects of the extent of cervical dilatation before emergency cerclage on neonatal outcomes and long-term follow-up results. This paper hypothesizes that the presence of cervical dilatation above 3 cm before cerclage will result in poor neonatal outcomes and long-term results.

## Materials and Methods

### Participants and Study Design

This retrospective clinical trial was conducted at a tertiary center, the Department of Obstetrics and Gynecology, between September 2020 and November 2020. The study was approved by the local ethics committee with IRB number 72867572-050.01.04-71556.

**Figure 1.**
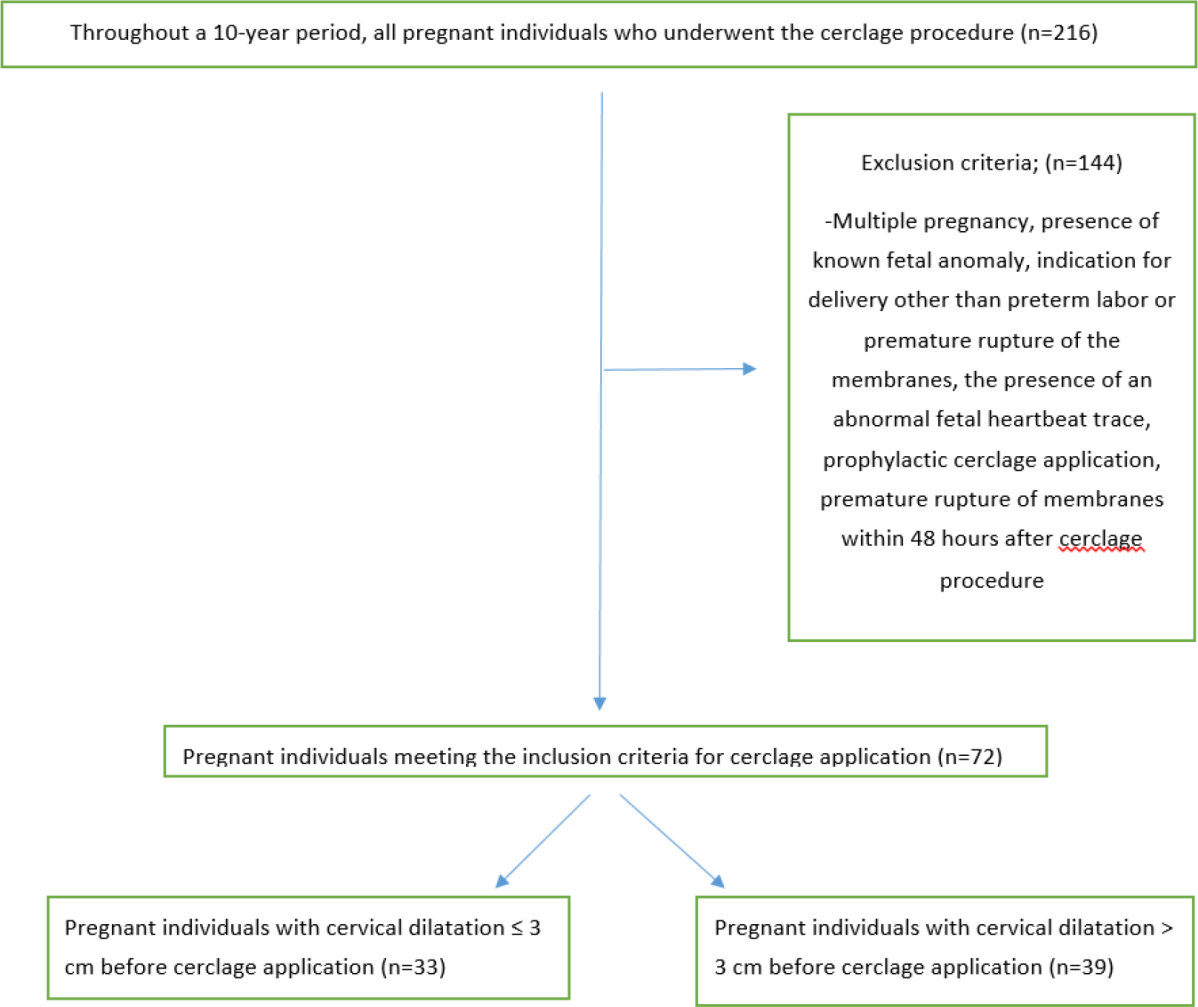
Participant selection and exclusion diagram.

All pregnant women who underwent cerclage for any reason within a ten-year period between January 2009 and January 2019 were screened. Data from 201 pregnant women who underwent cerclage were collected. Inclusion criteria were singleton pregnancy, intact amnion membrane, absence of clinical chorioamnionitis findings (fever with temperature >38.0°C, uterine tenderness, and foul-smelling discharge), no vaginal bleeding, and no uterine contractions. Exclusion criteria were multiple pregnancies, presence of known fetal anomaly, indication for delivery other than preterm labor or premature rupture of the membranes, the presence of an abnormal fetal heartbeat trace, prophylactic cerclage application, and premature rupture of membranes within 48 hours after the cerclage procedure (Figure 1).

The sample size calculation aimed to detect a 50% difference in births occurring before the 28th gestational week, with an alpha of 0.05, a beta of 0.2, and a power of 80%. It was determined that a sample size of 36 for both Group 1 and Group 2 would be sufficient.

Seventy-two pregnant women who underwent emergency cerclage procedures meeting the criteria were included in the study. The included patients were divided into two groups based on the cervical manual examination and/or transvaginal ultrasound examination performed by a gynecologist and obstetrician.

Group 1 consisted of thirty-three singleton pregnancies with cervical dilatation of 3 cm or less in cervical manual examination, or if there was no cervical dilation, cervical length of 5 mm or less in transvaginal ultrasound. Group 2 comprised thirty-nine singleton pregnancies with cervical dilatation above 3 cm in the cervical manual examination (Figure 1). The reason for determining 3 cm as the cutoff for cervical dilation was its objectivity in physical examination findings, as two fingers correspond to 3 cm dilation.

### Surgical Procedure

Surgical antibiotic prophylaxis was administered 30 minutes before each cerclage procedure. The procedure was performed using the McDonald technique with mersilene suture. Tocolytic treatment with indomethacin (100 mg rectal, then 4 × 25 mg oral) or nifedipine (30 mg/h, and then 6 × 10 mg) was administered to all pregnant women who underwent cerclage procedure for 48 hours post-surgery. Vaginal progesterone gel (90 mg) treatment was continued for all pregnant women until delivery.

### Evaluated Variables

Maternal variables included maternal age, parity, type of delivery, previous preterm delivery, previous cesarean delivery, presence of uterine anomaly, gestational day at cerclage application, gestational day at delivery, number of deliveries ≤28 weeks gestation (196 days), number of delivery between 28 weeks (196 days) and 34 weeks (238 days) gestation, number of delivery ≥34 weeks (238 days) gestations, need for blood transfusion at delivery, pregnancy prolongation from cerclage application-latency period, and white blood cell (WBC) value before cerclage application.

Participants were required to deliver at the hospital where the study was conducted to access the newborn records, which were available through the ‘e-Nabız’ system, allowing access to nationwide hospital admissions, examination records, laboratory results, radiological images, follow-ups, and discharge summaries. The requirement is for the ICD code to be entered by the relevant specialist physician as a ‘definitive diagnosis,’ with follow-ups continuing in the relevant specialties and/or with laboratory, radiological images, or physical examination findings of the relevant specialty clearly documented in the examination notes.

Neonatal variables included birth weight, umbilical cord blood pH, APGAR scores at 1st, 5th, and 10th minutes, the newborn intensive care unit (NICU) requirement, length of NICU stay (days), diagnosis of respiratory distress syndrome (RDS) based on the presence of clinical findings with a uniform ground-glass pattern on chest X-ray, neonatal sepsis confirmed by positive blood culture, having a follow-up for at least one year with a diagnosis of cerebral palsy diagnosis in a pediatric neurology clinic, diagnosis of neonatal jaundice with serum bilirubin level, follow-up at the ophthalmology clinic for retinopathy of prematurity, bronchopulmonary dysplasia, growth restriction, and malnutrition (below the 10th percentile), hydrocephalus, postnatal determination of congenital anomalies, hyperactivity disorder, intact survival (without any neurological or developmental delay in the long term), and mortality rate.

### Statistical Analysis

Data were expressed as mean ± standard deviation (SD) for continuous variables or frequencies (n) with percentages (%) for categorical variables. Shapiro-Wilk test was used to test the normality of data. Student’s t-test or Mann-Whitney test was employed to compare continuous variables, whereas the chi-square and Fisher’s exact tests were used for categorical data comparison. Logistic regression was applied to determine the independent variables affecting the outcome.

## Results

During the 10-year period, the results of 72 singleton pregnancies that underwent emergency cerclage procedures were further analyzed. Two groups were defined based on the amount of cervical dilatation in cervical examination. Thirty-three singleton pregnancies for Group 1 and thirty-nine for Group 2 were included.

**Figure 2.**
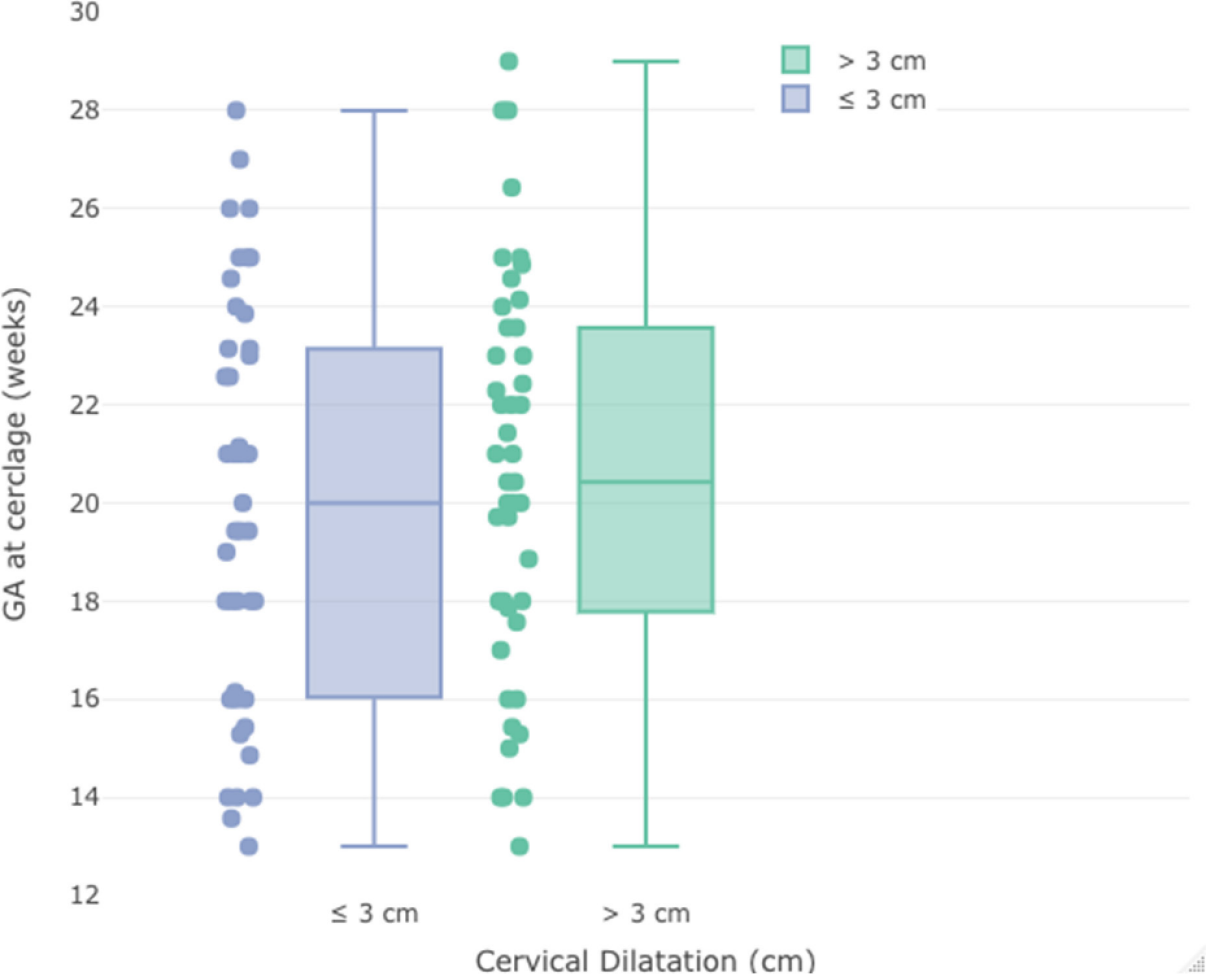
Distribution of gestational week at cerclage application to cervical dilatation before cerclage.

Characteristic and demographic parameters are provided in Table 1. At the time of the cerclage procedure for the two groups, the mean maternal age was 31.94 ± 7 and 34.38 ± 7.3, respectively, and the mean gestational day at cerclage application was 137 ± 30 and 144 ± 30, respectively. Mean maternal age and mean gestational day at the cerclage procedure were similar between groups (*p > 0.05*) (Figure 2). Preterm delivery history and the presence of previous cesarean section were statistically similar between the groups (*p > 0.05*). The WBC value before the cerclage application was similar between the two groups (*p = 0.98*).

**Figure 3.**
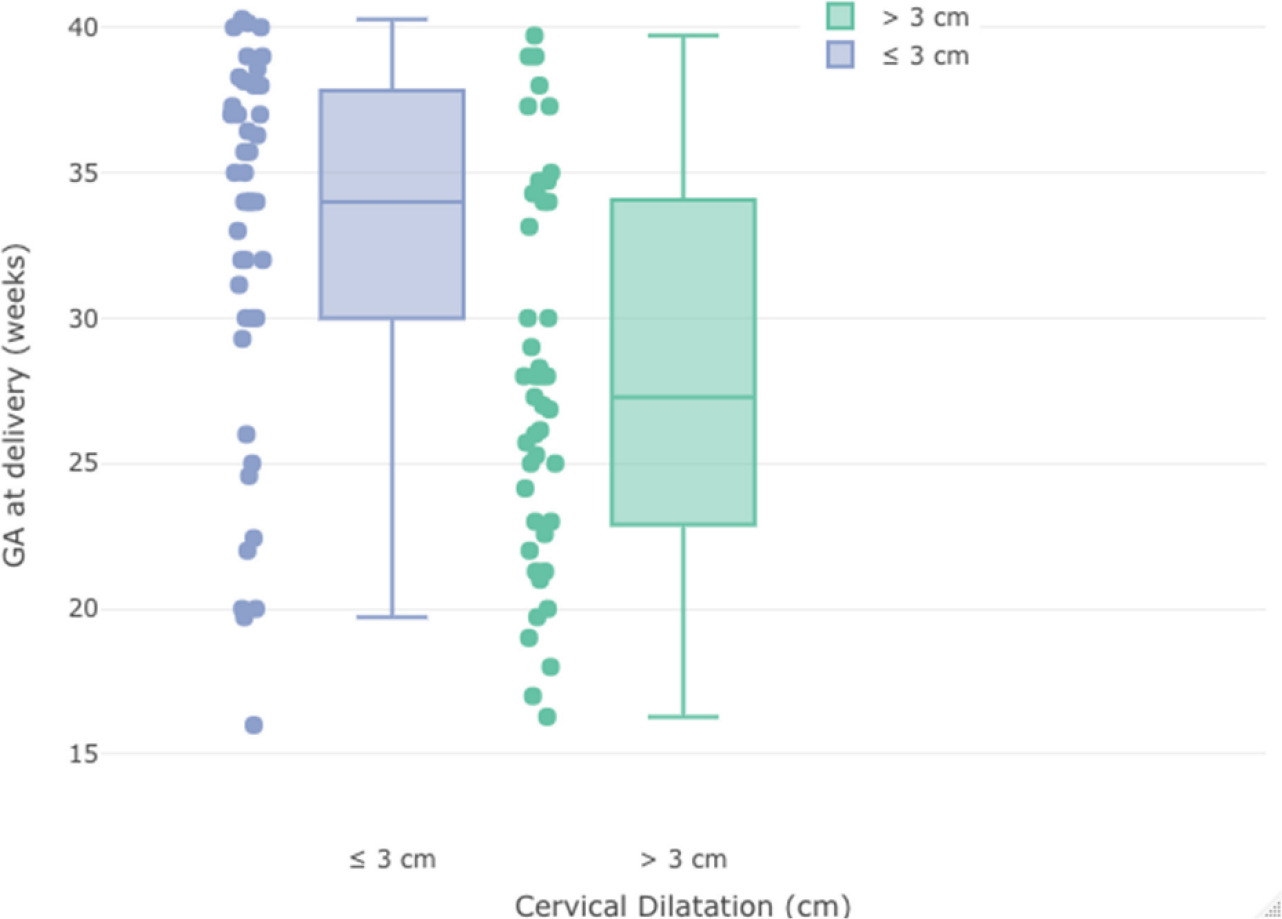
Distribution of gestational age at delivery according to cervical dilatation before cerclage application.

Gestation day at delivery for the two groups was 227 ± 51 and 195 ± 46, respectively (Figure 3). Group 2 had a statistically significant earlier delivery day than Group 1 (*p = 0.007*). In Group 1, the latency period was statistically longer than in Group 2 (*p = 0.005*). In Group 1, the delivery rate at ≤28 weeks gestation was lower, and the delivery rate at ≥34 weeks gestation was higher than in Group 2, respectively (*p = 0.0069, p = 0.0026*).

The comparison of early neonatal and long-term results is given in Table 2. Mean birth weights were 2159 ± 1153 and 1293 ± 1034, respectively. Group 1 had a higher birth weight than Group 2 (*p = 0.002*). Group 1 had higher first-minute APGAR scores than Group 2 (*p = 0.016*), but cord pH, 5th and 10th minute APGAR scores were not statistically different between groups (*p > 0.05*).

Neonatal outcomes were compared. Group 2 had poor early neonatal outcomes, including NICU requirement (*p = 0.006*), NICU days (*p = 0.006*), RDS (*p = 0.009*), neonatal jaundice (*p = 0.021*), and neonatal sepsis with positive blood culture (*p = 0.018*) for all these variables.

Long-term complications, including ROP, cerebral palsy, bronchopulmonary dysplasia, hydrocephalus, malnutrition, congenital anomaly, hyperactivity disorder, intact survival, and mortality parameters, were evaluated. ROP was 12% in Group 1 and 38% in Group 2. Group 2 had a statistically higher rate of ROP (*p = 0.009*). There was no significant difference between the groups for cerebral palsy, bronchopulmonary dysplasia, hydrocephalus, malnutrition, congenital anomaly, or hyperactivity disorder (follow-up for at least two years) (*p > 0.05*).

Intact survival rates were 48.4% and 23% for the groups. The intact survival rate in Group 2 was statistically significantly lower (*p = 0.013*). Mortality rates for the groups are 24.2% and 52.6%, respectively. The mortality rate in Group 2 was statistically significantly higher than Group 1 (*p = 0.015*).

Gestational age at delivery (p = 0.003), but not the degree of cervical dilatation at cerclage (p = 0.37), was independently associated with neonatal mortality (Table 3).

## Discussion

This study revealed that women with cervical dilatation >3 cm in manual examination before cerclage application experienced delivery at an earlier gestation age compared to those with cervical dilatation ≤3 cm. Cervical dilatation detected in manual examination before cerclage application emerged as an independent predictor of gestational age at delivery. In pregnancies with cervical insufficiency and cervical dilatation, cervical cerclage application has been shown to significantly prolong the gestation age at delivery compared to bed rest.^8,9^ On the other hand, the increase in cervical dilatation before cerclage application was found to be a correlation between the gestation age at delivery and earlier delivery time when cervical dilatation was ≥2 cm.^7^ Cervical cerclage application may prolong the delivery time, but the length of pregnancy prolongation decreases as cervical dilatation increases.

When WBC values were <14 (10^3^/µl) and cervical dilatation was <4 cm before cerclage application, more prolongation was obtained in the gestational period.^10^ Inflammatory markers are thought to have a role in predicting prognosis in pregnant women who underwent emergency cerclage.^11^ In this study, WBC value means were calculated and compared. On the contrary, WBC values were similar in the two groups separated according to cervical dilatation. Before the cerclage application, the amount of cervical dilatation is more important in predicting the prolongation of the gestational period.

In Group 2, cervical dilatation >3 cm before cerclage application resulted in high delivery rates at 28 weeks or below and low delivery rates at 34 weeks or above. This situation has resulted in lower birth weight and extreme prematurity. Group 1 had higher first-minute APGAR scores than Group 2 (*p = 0.016*), but cord pH, 5th and 10th minute APGAR scores were not statistically different between groups (*p > 0.05*). In another similar study, cervical dilatation >2 cm before cerclage application caused an increase in the value of 1 and 5 minutes APGAR score <7.^12^ This study showed no significant difference in neonatal vital parameters in the short term after delivery. Still, it was observed that more complications occurred in the medium and long term, especially in the group with a high rate of premature birth.

This study showed that if cervical dilatation is >3 cm in manual examination before cerclage application, early-middle neonatal results are much worse. In all parameters of NICU requirement, NICU days, RDS, neonatal jaundice, and neonatal sepsis with positive blood culture, poor results were obtained compared to patients with cervical dilatation ≤3 cm before cerclage application. The rate of preterm delivery increases proportionately to the increase in cervical dilatation determined by physical examination before the cervical cerclage procedure.^10^ Likewise, it was emphasized that the amount of cervical length influences the latent period.^13^ For this reason, assessing the condition of the cervix before cervical cerclage can be used to predict preterm birth and the resulting neonatal complications that may occur.

It has been reported that there is a 40% need for NICU after emergency cerclage application in which the membranes prolapse.^14^ However, this rate is much higher in this study, especially in the group with high cervical dilatation. This is due to the fact that birth occurred at much earlier gestational weeks in our study population.

It was stated that emergency cerclage application prolongs the delivery time, but cervical dilatation >3 cm increases the failure.^15^ Similarly, prolongation of delivery time was less in patients with cervical dilatation >3 cm, resulting in delivery at an earlier gestational week. Likewise, cervical dilatation >3 cm before cerclage application resulted in lower birth weight. Earlier delivery weeks and lower birth weights cause an increase in early neonatal complications.

In this study, when the groups were compared for long-term complications, Group 2 had higher mortality and lower intact survival rates. It has been shown before that the cervical dilatation threshold was assumed to be 2 cm, and they investigated the effect of emergency cerclage application on neonatal outcomes. Neonatal death rates at birth were reported as 9.5% in the group with ≤2 cm cervical dilatation and 43.8% in the group with >2 cm cervical dilatation in their study.^12^ In this study, mortality rates were 24.2% and 52.6%, respectively. Contrarily, higher mortality rates were found in our data. This was because the mortality from birth to the present was calculated. Deaths due to prematurity complications in the first years of life were included in the mortality rate, especially in Group 2. If the follow-up period is extended, a higher mortality rate can be obtained. Therefore, intact survival rates may be a better indicator for predicting long-term complications. Intact survival rate is important in evaluating neonatal morbidities. It is clearly stated in the article that it evaluates long-term morbidities, especially after extreme preterm birth. It has been emphasized that in the long term, 42% of children born between 24-28 weeks of gestation require health care due to premature birth.^16^ Intact survival rates for the groups are 48.4% and 23%, respectively. If the cervical dilatation before cerclage is >3 cm, very low intact survival rates are expected.

The frequency of ROP was 12% and 38% for the groups, respectively. Group 2 had a statistically significantly higher ROP rate than Group 1. The incidence of ROP increases in association with preterm gestation and low birth weight.^17^ Emphasis is placed on delaying premature birth and the use of cervical cerclage and vaginal progesterone to prevent the development of ROP.^18^ In the United States, the incidence of ROP was 0.17% for the 8-year period and 15.58% among premature births.^19^ The reason for the determination of ROP rates much higher than the ROP incidence found in premature births in Group 2 is due to the extremely preterm gestational age at delivery and very low birth weight.

The rate of cerebral palsy (CP) was similar in both groups, 8% and 9%, respectively. The rate of cerebral palsy was defined as 0.2% in the population. Many risk factors have been identified for CP, such as low birth weight and intrauterine infection.^20^ In the subgroup analysis performed, cerebral palsy was associated with a low gestational week at delivery independent of cerclage application week and cervical dilatation. The amount of cervical dilatation cannot be used to predict cerebral palsy, and emergency cerclage application increases the CP rate. In a previous study, high rates of CP were reported after applying cervical cerclage in patients with prolapsed membranes to the vagina.^5^ Therefore, considering the frequency of CP in the normal population, the frequency of CP increased in pregnancies that underwent emergency cerclage.

This study has some limitations. The retrospective design of the study is a significant limitation. Conducting an archive review over a long period of 10 years ensured a high number of patients, but the fact that different specialists performed the cerclage procedure led to heterogeneity.

## Conclusion

Advanced cervical dilatation (>3 cm) during physical examination-indicated cerclage in singleton pregnancies is associated with earlier delivery, leading to increased neonatal morbidity and mortality when compared with pregnancies having lesser degrees of cervical dilatation at cerclage, although short-term poor neurological outcomes seem comparable. Considering the higher likelihood of early delivery after cerclage compared to low-risk pregnancies, the potential for short and long-term neonatal adverse outcomes should be taken into consideration. Especially in cases where there is a high degree of cervical dilation before the cerclage procedure, it is crucial to inform these pregnant individuals about these neonatal adverse outcomes. The findings need to be supported by studies conducted with larger populations.

## Ethics Committee Approval

The study was performed per the ethical standards for human research established by the Declaration of Helsinki and Good Clinical Practice guidelines and was approved by the Suleyman Demirel University Faculty of Medicine Ethics Committee with IRB number 72867572-050.01.04-71556.

## Informed Consent

All patients provided written informed for the application of this technique.

## Conflict of Interest

The authors report no conflict of interest.

## Figures and Tables

**Table 1.** Comparison of characteristic and demographic parameters between groups.

	**Cervical dilatation ≤3 cm (n = 33)**	**Cervical dilatation >3 cm (n = 39)**	p-value (*)
Maternal age (years)	31.94 ± 7.0	34.38 ± 7.3	0.15
Parity	0.79 ± 0.8	0.81 ± 1.46	0.25
Type of delivery (cesarean rate)	20/33 (60.6%)	17/39 (43.6%)	0.15
Previous preterm delivery	1/33	1/39	0.68
Previous cesarean delivery	7/29 (24.1%)	4/31 (12.9%)	0.26
WBC value before cerclage application (10^3^/µl)	11.4 ± 4.0	11.4 ± 2.8	0.98
Gestational day at cerclage application	137 ± 30	144 ± 30	0.32
Gestational day at delivery	227 ± 51	195 ± 46	0.007*
Pregnancy prolongation from cerclage application-latency period (days)	90 ± 55	52 ± 54	0.005*
No. of delivery ≤ 28 weeks’gestations			
(196 days)	9/33 (27.3%)	23/39 (59%)	0.0069*
No. of delivery between 28 weeks			
(196 days)-34 weeks (238 days)’ gestations	5/33 (15.2%)	7/39 (17.9%)	>0.05
No. of delivery ≥34 weeks’ gestations			
(238 days)	19/33 (57.6%)	9/39 (23.1%)	0.0026*
Need for blood transfusion at delivery	4/21 (19%)	3/31 (9.7%)	0.33

WBC, White blood cell.

*Show that the p-value is <0.05

**Table 2.** Comparison of early and late neonatal outcomes between groups.

	**Cervical dilatation ≤3 cm (n = 33)**	**Cervikal dilatation >3 cm (n = 39)**	p-value (*)
Birth weight (g)	2159 ± 1153	1293 ± 1034	0.002*
Cord blood pH	7.32 ± 0.04	7.30 ± 0.13	0.63
APGAR 1. Min	7.81 ± 0.51	6.86 ± 1.65	0.016*
APGAR 5. Min	8.81 ± 0.68	8.33 ± 1.23	0.095
APGAR 10. Min	9.90 ± 0.44	9.45 ± 1.23	0.082
NICU requirement	10/27 (37%)	20/27 (74%)	0.006*
Length of NICU stay (days)	10 ± 24	26 ± 37	0.006*
RDS	5/25 (20%)	15/25 (60%)	0.009*
Neonatal jaundice	6/25 (24%)	14/25 (56%)	0.021*
Neonatal sepsis with positive blood culture	5/25 (20%)	14/25 (56%)	0.018*
ROP	3/25 (12%)	8/21 (38%)	0.009*
Cerebral palsy	2/25 (8%)	2/22 (9%)	0.64
Bronchopulmonary dysplasia	1/24 (4.1%)	3/22 (13.6%)	0.27
Hydrocephalus	0/24 (0%)	3/21 (14.2%)	0.09
Malnutrition	2/24 (8.3%)	3/21 (14.2%)	0.43
Congenital anomaly	0/24 (0%)	1/21 (4.7%)	0.46
Hyperactivity disorder	1/24 (4.1%)	1/21 (4.7%)	0.72
Intact survival	16/33 (48.4%)	9/39 (23%)	0.013*
Mortality	8/33 (24.2%)	20/38 (52.6%)	0.015*

NICU, The newborn intensive care unit; RDS, Respiratory distress syndrome; ROP, Retinopathy of prematurity.

*p-value is <0.05

**Table 3.** Logistic regression model table for mortality outcomes.

	**B**	**SE.**	**Wald**	**p**	**Exp(B)**
**Cervical dilatation**	−1.199	1.341	0.799	0.37	0.302
**Gestational day at delivery**	0.124	0.042	4.54	0.003*	1.132

*: p < 0.05 Two-category logistic regression.
